# Editorial: Targeted alpha particle therapy in oncology

**DOI:** 10.3389/fmed.2023.1165747

**Published:** 2023-03-07

**Authors:** Øyvind Sverre Bruland, Roy Hartvig Larsen, Richard Paul Baum, Asta Juzeniene

**Affiliations:** ^1^Department of Oncology, The Norwegian Radium Hospital, Oslo University Hospital, Oslo, Norway; ^2^Institute for Clinical Medicine, University of Oslo, Oslo, Norway; ^3^Sciencons AS, Oslo, Norway; ^4^Curanosticum Wiesbaden-Frankfurt, Center for Advanced Radiomolecular Precision Oncology, Wiesbaden, Germany; ^5^Department of Radiation Biology, Institute for Cancer Research, The Norwegian Radium Hospital, Oslo University Hospital, Oslo, Norway; ^6^Department of Physics, University of Oslo, Oslo, Norway

**Keywords:** actinium-225, astatine-211, lead-212, radium-223, radium-224, thorium-227, targeted alpha therapy (TAT), targeted radionuclide therapy (TRT)

Targeted radionuclide therapy (TRT), also known as molecular radiotherapy, targeted radiotherapy, or radiotheranostics, is a rapidly developing area with important recent breakthroughs (1–3). It aims to treat disseminated cancer, the main clinical challenge in oncology (4, 5). TRT is based on personalized patient selection using molecular imaging to verify the presence of a biologic target either on the cancer cell surface or in vascular and/or stromal elements of metastases. The only approved alpha-emitting radiopharmaceutical is Xofigo (^223^RaCl_2_, approved in 2013). The recent approval of beta-emitting ^177^Lu-PSMA-617 (Pluctivo, approved in 2022) for the treatment of metastatic castration-resistant prostate cancer (mCRPC) expressing prostate-specific membrane antigen (PSMA), and of ^177^Lu-DOTATATE (Lutathera, approved by EMA in 2018) for therapy of somatostatin receptor positive neuroendocrine tumors (NETs) will clearly shift TRT into the mainstream of cancer treatment. Nevertheless, some patients either do not respond, or, following initially good response, develop resistance to ^177^Lu-based therapies, in spite of sufficient expression of target proteins on cancer cell surfaces (6, 7). Many preclinical and clinical trials have demonstrated that alpha-particle-emitting radiopharmaceuticals, due to their physical properties, high linear energy transfer, and short range in tissue relative to beta-emissions, are emerging as a promising approach for cancer treatment (8–11); they can also directly kill hypoxic or radio- and chemo-resistant cancer cells.

The goal of this Research Topic is to describe the development of novel alpha-emitting radiopharmaceuticals for different cancers, recent preclinical, completed, and ongoing clinical trials of targeted alpha-particle therapy (TAT) alone or in combination, dosimetry, safety, challenges related to supply and availability of suitable alpha-emitting radionuclides, as well as some future perspectives. This Research Topic includes 16 articles focusing on original research (four articles), reviews on different aspects of TAT (9 articles), ongoing clinical trials (one article), study protocols (one article), and hypotheses and theories (one article). Key opinion leaders, medical doctors, and scientists from Australia, Belgium, France, Germany, Poland, Norway, Singapore, Sweden, Switzerland, the United Kingdom, and the United States have contributed to this Research Topic.

Only a few radionuclides, namely, ^225^Ac, ^211^At, ^212^Bi, ^212^Pb/^213^Bi, ^224^Ra, ^223^Ra, and ^227^Th, are of interest for TAT. In this Research Topic, the challenges and benefits of these radionuclides are reviewed.

Bone-seeking ^223^RaCl_2_ is approved for patients with mCRPC and dominant osteoblastic skeletal metastases. Attempts to complex ^223^Ra to cancer cell–targeting moieties have been unsuccessful. Some researchers and medical doctors speculate that ^223^RaCl_2_ will be less used after the approval of cancer cell–targeting ^177^Lu-PSMA-617. In this Research Topic, O'Sullivan et al., Sartor and Baghian, and Kostos et al. discuss the potential of ^223^Ra and why it seems underutilized. Despite the survival rate benefit of cancer cell–targeting ^177^Lu-PSMA-617, responses for many patients with mCRPC are not long-term, and almost all patients will subsequently develop progressive disease. Sartor and Baghian reviewed the rapidly developing, most promising radiopharmaceuticals, including ^225^Ac-, ^212^Pb-, and ^227^Th-labeled PSMA-binding ligands and their future. Tandem therapies combining beta and alpha radiopharmaceuticals are also presented. Kostos et al. present a protocol for the clinical study of AlphaBet, combining a bone-specific alpha-emitter ^223^Ra with the beta-emitter ^177^Lu-PSMA-I&T, for the eradication of micrometastatic osseous disease, since the bone marrow is the most common site of cancer progression. Micrometastases in the skeleton likely receive an inadequate dose of radiation as the emitted beta-particles from ^177^Lu travel an average distance of 0.7 mm in soft tissue, well-beyond the diameter of micrometastases. Bone-seeking ^223^RaCl_2_ can be used alone or in combination with gemcitabine or denosumab for osteoblastic osteosarcoma treatment, as described by Anderson et al.. Unfortunately, not all areas of osteosarcoma lesions are osteoblastic. In such cases, TAT with ^225^Ac or ^227^Th targeting IGF1R or Her-2 overexpressed in osteosarcoma may become efficient treatment for osteosarcoma (NCT03746431 and NCT04147819).

In TAT, radionuclides are delivered to cancer cells through a wide variety of formulations such as radiolabeled antibodies, peptides, or small molecules. A recent strategy incorporates ^224^Ra into CaCO_3_ microparticles (Radspherin^®^), designed as a treatment of the remaining peritoneal micrometastasis in ovarian and colorectal cancer after complete cytroreductive surgery, as a means to decrease ^224^Ra and its daughters' redistribution from the peritoneal cavity (12). The goal of the product is to generate an alpha particle radiation field on the surfaces and liquid volumes of the peritoneal cavity. Wouters et al. have shown the therapeutic efficacy of ^224^Ra-CaCO_3_ in a mouse model of ovarian cancer, and the possibility for safe sequential administration using several chemotherapy regimens commonly employed in patients. Larsen et al. report the first study on Radspherin for peritoneal metastasis of colorectal cancer in 23 patients. Biodistribution studies demonstrated that Radspherin was distributed peritoneally. Dose-limiting toxicity was not reached. The safety issues of Radspherin and the level of radiation exposure from the patients to surrounding people were described by Grønningsæter et al.. It was concluded that there was no need for any restrictions or precautions due to external exposure.

The dosimetry and radiation risk–related aspects of ^224^Ra and ^223^Ra have been discussed by Lassmann and Eberlein.

A novel dual alpha technology with potentially broad therapeutic applications (new generator and radiopharmaceuticals), comprising ^224^Ra for targeting the osteoblastic stroma of bone metastases and the chelated-conjugate daughter ^212^Pb for selective binding to tumor cells, has been proposed by Juzeniene et al. and Tornes et al. in this Research Topic.

Shi et al. reviewed the strengths, weakness, and the present and future of ^225^Ac-labeled somatostatin receptor agonists and antagonists in preclinical and clinical applications for NETs.

Karlsson et al. summarized preclinical and clinical studies on ^227^Th-conjugates for various cancer types. The authors also discussed the feasibility of using ^227^Th-conjugates in combination with other therapies. The potential of the combination of ^227^Th-conjugates and PD-1 check-point inhibitors in preclinical models was demonstrated by Berg-Larsen et al..

In a comprehensive review, Albertsson et al. discussed completed and ongoing clinical trials of different ^211^At-conjugates.

Kunikowska et al. provide an overview of strategies for the local treatment of primary and secondary glioblastomas using ^213^Bi, ^225^Ac, and ^211^At. Antibodies targeting the extracellular matrix protein tenascin and substance P targeting the neurokinin type-1 receptor overexpressed in glioblastomas were discussed as targeting moieties for TAT.

It is crucial to select novel target molecules that are expressed in various types of cancers, and preferentially develop radiopharmaceuticals both for imaging and therapy, allowing a theranostic approach. However, not all targets are suitable for TAT; some are useful only for imaging. The biological effect of TAT depends on the absorbed dose, which is related to the “area under the curve”; the biological half-life at tumor sites and normal tissues, matched with the physical half-life of the given alpha emitter. Additionally, dosimetry calculations of TAT are challenging, since alpha particles have short ranges (<100 μm) that may provide heterogeneous irradiation and their daughters may have different pharmacokinetic profiles and chemical properties.

TAT is one of the most rapidly growing fields in the management of different types of cancer, and many radiopharmaceuticals are already in clinical trials. Commercial and business aspects of alpha radioligands have been discussed by Ostuni and Taylor.

We hope that this Research Topic on TAT will stimulate more research and clinical trials in this field.

## Author contributions

All authors listed have made a substantial, direct, and intellectual contribution to the work and approved it for publication.

## References

[1] Herrmann K Schwaiger M Lewis JS Solomon SB McNeil BJ Baumann M. Radiotheranostics: a roadmap for future development. Lancet Oncol. (2020) 21:e146–e56. 10.1016/S1470-2045(19)30821-6 32135118 PMC7367151

[2] Dolgin E. Radioactive drugs emerge from the shadows to storm the market. Nat Biotechnol. (2018) 36:1125–7. 10.1038/nbt1218-1125 30520873

[3] Dolgin E. Drugmakers go nuclear, continuing push into radiopharmaceuticals. Nat Biotechnol. (2021) 39:647–9. 10.1038/s41587-021-00954-z 34113040

[4] Dillekås H Rogers MS Straume O. Are 90% of deaths from cancer caused by metastases? Cancer Med. (2019) 8:5574–6. 10.1002/cam4.2474 31397113 PMC6745820

[5] Coleman R Hadji P Body JJ Santini D Chow E Terpos E. Bone health in cancer: ESMO clinical practice guidelines. Ann Oncol. (2020) 31:1650–63. 10.1016/j.annonc.2020.07.019 32801018

[6] Kratochwil C Bruchertseifer F Giesel FL Weis M Verburg FA Mottaghy F. ^225^Ac-PSMA-617 for PSMA-targeted α-radiation therapy of metastatic castration-resistant prostate cancer. J Nucl Med. (2016) 57:1941–4. 10.2967/jnumed.116.178673 27390158

[7] Ballal S Yadav MP Tripathi M Sahoo RK Bal C. Survival outcomes in metastatic gastroenteropancreatic neuroendocrine tumor patients receiving concomitant (225)Ac-DOTATATE targeted alpha therapy and capecitabine: a real-world scenario management based long-term outcome study. J Nucl Med. (2022). 10.2967/jnumed.122.264043 35863893

[8] Pouget JP Constanzo J. Revisiting the radiobiology of targeted alpha therapy. Front Med. (2021) 8:692436. 10.3389/fmed.2021.692436 34386508 PMC8353448

[9] Makvandi M Dupis E Engle JW Nortier FM Fassbender ME Simon S. Alpha-emitters and targeted alpha therapy in oncology: from basic science to clinical investigations. Target Oncol. (2018) 13:189–203. 10.1007/s11523-018-0550-9 29423595

[10] Nelson BJB Andersson JD Wuest F. Targeted alpha therapy: progress in radionuclide production, radiochemistry, and applications. Pharmaceutics. (2020) 13:49. 10.3390/pharmaceutics13010049 33396374 PMC7824049

[11] Eychenne R Chérel M Haddad F Guérard F Gestin JF. Overview of the most promising radionuclides for targeted alpha therapy: the “hopeful eight”. Pharmaceutics. (2021) 13:906. 10.3390/pharmaceutics13060906 34207408 PMC8234975

[12] Li RG Lindland K Tonstad SK Bønsdorff TB Juzeniene A Westrøm S. Improved formulation of (224)Ra-labeled calcium carbonate microparticles by surface layer encapsulation and addition of EDTMP. Pharmaceutics. (2021) 13:634. 10.3390/pharmaceutics13050634 33946852 PMC8145685

